# Antimicrobial policies in animals and human health

**DOI:** 10.1186/s13690-017-0231-7

**Published:** 2017-10-19

**Authors:** Boudewijn Catry

**Affiliations:** Healthcare-Associated Infections & Antimicrobial Resistance, Operational Directorate of Public Health and Surveillance, Scientific Institute of Public Health (WIV-ISP), J. Wytsmanstraat 14, 1050 Brussels, Belgium

## Abstract

This invited commentary aims to highlight 3 recommendations to rationalize antimicrobial consumption in livestock, in order to minimize the spread of resistant bacteria and simultaneously safeguarding animal and public health.

## Background

### The early recognition of antimicrobial resistance hazards in livestock

Nearly all antimicrobial compounds used in human have a counterpart in veterinary medicine [1]. In livestock, the majority of antimicrobial agents are currently given largely for the prevention of disease by oral group treatments. Historically antimicrobial agents were given from after World War II onwards to otherwise healthy food producing animals to enhance growth, increase feed conversion and decrease waste and (zoonotic) infections. Due to different reports in the 1960’s on zoonotic infections with antimicrobial resistance linked with the use of antimicrobial agents on the respective farms, the use of these agents as antimicrobial growth promoter (AMGP) has been a source of intense debate. Based on the precautionary principle, this resulted in a gradual restriction of this particular AMGP use in different European countries and a total ban from January 2006 onwards in the entire EU.

### Livestock associated MRSA

Simultaneously with this stepwise decrease of consumption of antimicrobial agents as growth promoters, different EU countries have monitored an increase in the use of therapeutic compounds. Could this unforeseen consequence of the ban on AMGP - paradoxically - be associated with the occurrence of multidrug resistant organisms in livestock? Some evidence suggests this is might be the case, e.g. the occurrence of Methicillin resistant *Staphylococcus aureus* (MRSA) in European livestock. One of the most administered classes of drugs ‘therapeutically’ given is tetracyclines. Tetracyclines are given mostly by the oral route to contain outbreaks of respiratory and digestive disorders (metaphylaxis). In livestock, their use has been associated with tetracycline resistance levels in the commensal gut microbiota [2]. In man, MRSA has been a major nosocomial burden since the 1990’s worldwide, in particular in acute care hospitals and then followed by long term care facilities. A particular clone has been discovered since 2003 in livestock, termed livestock associated MRSA (clonal complex 398), but its occurrence in human medicine remains fairly low. The vast majority of reports on MRSA in livestock (CC398) document a presence of 100% tetracycline resistance [3], tetracycline use might have enhance the widespread of these organisms. One report associated the use of group treatments with the occurrence of MRSA CC398 in swine [4]. Because CC398 are also often resistant for other antimicrobial agents, [potentiated sulphonamides (sulfa-TMP; cotrimoxazole), beta-lactam antibiotics, aminoglycosides, macrolides-lincosamides-streptogramines antibiotics (MLS), fluoroquinolones] the administration of these antimicrobials also can contribute to the selection for the livestock-associated MRSA. The fact that this MRSA lineage has been found in comparable amounts in other countries in which AMGP are still used e.g. Canada [5], contradicts the simple but paradoxical statement that the ban of AMGP is associated with the occurrence of CC398. Further research is slowed down by a decrease of MRSA infections in human medicine during the last decade (https://www.cdc.gov/drugresistance/biggest_threats.html). The decrease of MRSA in man is thought to be the result of several factors: active screening, stewardship, rapid diagnostics, isolation procedures, decontamination and improved hand hygiene among healthcare workers. Other pathogens originating from the digestive tract, such as intrinsic resistant *Clostridium difficile* and multidrug resistant Gram-negatives are now easily spreading among *fragile* livestock and their derived products. This is worrisome since antimicrobial consumption there remains high given the intensive production systems.

### Other Darwinian selections

Some researchers ascribe the aforementioned elevated consumption of therapeutic agents during the last decade - and thus the period of the stepwise ban of AMGPs - to new diseases entities like clostridial infections in livestock [6]. The majority of AMGPs have a Gram positive and anaerobic spectrum, and resistance has always been found to be low in animal pathogenic *Clostridium* species [7]. An efficient inhibition of their multiplication could have been possible under a regimen of AMGP. Thus, it is possible that the removal of this selection pressure was in favour of these organisms, including pathogenic species. According to this scenario, a temporal raise of some disease entities in livestock will be triggered leading to changes in antimicrobial consumption. After a certain or steep rise in antimicrobial consumption this could level off and eventually decrease, due to breed selection of animals that are less susceptible for these infections. This scenario probably has taken place in Sweden as in detail described by Martin Wierup [8]. Sweden is a pioneer with regard to the ban of AMGP and executed it already in 1986. After a total replacement of a certain animal herd, a similar Darwinian selection process will take place, with adaptations to the new management and environmental conditions [8, 9]. Of notice, such total replacements have been present during the last decades for many diseases, including BSE, classical swine fever, avian Influenza, Q-fever. Transported and imported animals might have introduced foreign microbiomes that respond differently on all introduced antimicrobial policies.

### Ranking, ranking, ranking

There have been numerous attempts to encourage the judicious use of antimicrobials in veterinary medicine [10]. One major issue addressed in such guidelines is to preserve the 3rd generation cephalosporins and fluoroquinolones, the so-called ‘reserve drugs’ important for human use. One must however keep in mind that every use of antimicrobial agents will lead to a selection pressure in favour of resistant subpopulations [11], albeit through mutations or by favouring organisms with horizontally transferable elements [12, 13]. After Darwinian selection, multidrug resistance mechanisms [14] evoke an extremely efficient spread of antimicrobial resistance by combining cross-resistance (resistance to structurally related compounds) and co-selection (resistance to structurally unrelated compounds). It has been demonstrated that the use of tetracyclines has been involved in the emergence of LA-MRSA (ST398). Thus one could conclude that this classification of tetracyclines as a ‘*no harm* drug’ did not effectively limit the spread of multidrug resistant organisms. Veterinary practitioners might have been prescribing older antimicrobial agents (tetracyclines, MLS compounds, cotrimoxazole i.e. potentiated sulphonamides) under the assumption that these are/were no critical drugs. As a consequence veterinarians mistakenly could have thought that their application would not worsen the antimicrobial resistance situation from a public health point of view.

Polymyxins are currently the next in line to have received - as antimicrobial class - the ‘preserved status’ from a public health point of view. Colistin is a member of the polymyxin family and for toxicity reasons seldom used in human medicine, despite its availability for decades. Given that colistin is poorly absorbed following oral intake in animals, colistin residues were not present in the derived food products. Resistance in target pathogens was never reported. Farmers and veterinarians thus were convinced it was relatively safe in animals.

Slowly resistance in Gram-negatives to beta-lactams arose in human medicine, respectively relatively slowly for amoxicillin-clavulanic acid, more rapid for different generations of cephalosporins and during 2010–2015 relatively fast for carbapenems. Within these five short years, colistin went from a product that was almost never used in human medicine, to a product that was one of the last resorts for Gram-negative infections. Cystic fibrosis patients were among the first casualties. Veterinarians and farmers were unaware of this shift in the therapeutic value of polymyxins in human medicine, and continued to administer colistin with few concerns in relation to antimicrobial resistance.

Colistin is still abundantly used in livestock, and honourable attempts to reduce this have been launched, e.g. at the European level (Updated advice on the use of colistin products in animals within the European Union: development of resistance and possible impact on human and animal health EMA/CVMP/CHMP/231573/2016). WHO now also has upgraded the critical importance of colistin to the highest level following the (retrospective) discovery of horizontally transferable colistin resistance. In contrast, and remarkable, the WHO has downgraded the importance of pleuromutulins from a public health point of view (http://www.who.int/foodsafety/publications/antimicrobials-fifth/en/). It is only a matter of time before the further spread of resistance and cross-resistance in human medicine will soon demand a revision of the list of CIAs (critically important antimicrobials).

I conclude that given the continuous drift of resistance hazards following the Darwinian selection pressure - which has been recently documented extremely visible and persuasive (https://www.youtube.com/watch?v=plVk4NVIUh8) -, it might therefore be more in place not to focus on certain antimicrobial classes or agents, but more in general on the prevention of *overall* antimicrobial resistance while maintaining *overall* clinical efficacy. In mammals *overall*. Some examples are given in the next paragraphs.

## Foreground

### In feed, in water, in milk

The aforementioned term ‘therapeutic’ consumption needs to be further put in perspective. Many treated animals are not sick at the initiation of the antimicrobial therapy. Prevention of respiratory and digestive disorders can be done by treating the animals in a known risk period during the production cycle, termed prophylaxis. In addition, it is currently allowed, to treat healthy animals sharing the same space (barn, stable, pen) with diseased individuals. This use is termed metaphylaxis. Many experts consider both prophylaxis and metaphylaxis necessary in a modern livestock industry and both systems are common practice in the majority of intensively reared animals like broilers, fattening pigs and veal calves. They are *fragile* – requiring specific care - given their age and high stock densities. Of particular importance, prophylaxis (e.g. starter rations) and metaphylaxis largely resemble the use of AMGP: to large group of intensively reared animals antimicrobial agents are given through the feed or drinking water. The three major deviations between AMGP and oral metaphylaxis are the dose, the duration of therapy, and the fact a subset of the treated animal populations shows signs of epidemic bacterial infections or is expected to become sick in the near future. However, prophylactic and metaphylactic antimicrobial use can be given at fairly lower doses than registered for curative use, largely exceeding the leaflet duration proposal, and microbiological examinations are mostly not performed [15].

Giving antimicrobial agents through the feed has major economic and ergonomic advantages, and no residues at an injection site. Approximately 90% of antimicrobial consumption in livestock is given by the oral route [15]. Yet prior to the entry of the compound at the site of infection, several resistance selection pressures are pronounced compared to e.g. injectable formulations. First, during the preparation e.g. in the feed or milk diet/drinking water, which is in addition highly variable since the final obtained concentration depends on water hardness, pH, temperature and/or U.V. light [16], often hard to control for. Since the same machines are used between different therapies, drag over of antimicrobial residues also is possible. In addition, in sick animals or due to the hierarchy structure in a flock – the amount administered cannot always be guaranteed. Secondary, the selection pressure exerted on the skin and in the digestive tract, starting from the oropharynx and ending in the faeces and by consequence the environment, is well documented for different antimicrobial agents in animals and humans [17, 18]. Parenteral formulations need to be actively excreted in the gut when similar resistance selection pressure can be expected. Then efficient resistance selection is pronounced also after injection [19].

### In syringe

Whereas many concepts, in addition or related to the above concept of ‘preserve reserved drugs’ have been tried out (e.g. cycling/rotation therapy, combination therapy), the general consensus guideline to optimise antimicrobial drug use is both in human and veterinary medicine to give a high dose for a minimum period of time [20]. This theory can now be found in the current veterinary medicine formulary too. One major example is the rise of one shot injectable antimicrobials registered mainly for respiratory livestock diseases. The rationale behind this concept is that an elevation of the concentration avoids the rise of resistant subpopulations. Bacteria are not only inhibited (bacteriostatic) but also killed (bacteriocidal). Although the theory is mainly based on aminoglycosides and quinolones and so far restricted to mutational resistance (not horizontal gene transfer; HGT), the concept also seems applicable for antimicrobial agents that have not a pronounced concentration dependent action and in which HGT is of importance [21].

The concept of high short dosage relies on a maximum amount of pathogenic bacteria present at the site of infection. Hereby, one assumes that at the site of infection approximately 10^10^ pathogenic bacteria are present and two sequential mutations at an estimated frequency of 1 × 10^−9^ are avoided by an elevation of the concentration [20]. One must consider that this maximum number of organisms is exceeded for commensal bacteria in every (!) ml of luminal content in the lower digestive tract alone [22, 23]. The routine practice of orally given group metaphylaxis, often for up to several weeks, is at right angles to the short high dosage guideline. The difference between the selection and spread of resistance between oral and injectable formulations in the faecal flora alone is extremely high (log4 which means 10,000 fold) [9]. Therefore mass oral group medication should be discouraged.

## Hygiene and conflict of interests

The influence of the hygiene protocol in between production rounds in livestock on the occurrence and maintenance of resistance has been investigated. Alas unsuccessful so far [24, 25]. In the authors’ opinion, thermal cleaning, the products used, the duration of drying and the period in which no animals are kept (the stand alone period) needs further urgent evaluation and improvement. Steaming, U.V., and vaporisation systems need to be considered, despite that mechanical cleaning, cleansing and drying are by far the most critical decontamination steps.

Generic compounds are becoming more and more available because patent protection to the original developer has expired. In many case, this will lower the price for both the original brand name and the generic compound. A decrease in price can increase the administration of compounds. Many compounds used for the treatment of large animals groups can be found in generic form and the rise of companies specialised in the merchandising of generic compounds might have influenced the consumption patterns.

In addition the income of some veterinarians partly or substantially can rely on the figure sales of medication. This also can drive antimicrobial prescription patterns and therefore the selection pressure exerted in the herds (EMA and EFSA Joint Scientific Opinion on measures to reduce the need to use antimicrobial agents in animal husbandry in the European Union, and the resulting impacts on food safety RONAFA - [EMA/CVMP/570771/2015]). We all have ambition and relatives and one cannot neglect some conflicts of interests.

## Recommendations

Taking the current views on rational antimicrobial therapy and knowledge on resistance into account, one could summarize a judicious veterinary use as follows:

1. Minimise the number of herds, animals and commensal bacteria exposed by avoiding oral group medications and separating sick from healthy animals.
2. Maximise the number of pathogenic bacteria exposed by applying individual short high dosages as local as possible to the infection site.
3. Replace oral group medication by appropriate infection control measures in between two consecutive production cycles, currently an idle opportunity to break the vicious spiral of multi-resistant organisms.

Studies demonstrating the applicability and resistance effects of these recommendations, without jeopardising animal health are needed and the author is optimistic: some projects are on-going.

## Epilogue

As always, changing a common practice like oral group medications from 1 day to another will have an effect on ecological and bacteriological balances including the pathogens and by consequence on animal performance. Close veterinary surveillance sustained by microbiological examinations is highly encouraged. Given the fast evolution in diagnostics [26], a rapid herd screening for virulent pathogens by (e.g.) a mobile spectrometric lab, no longer seems science fiction. It will help to lower the use of empiric large spectrum antimicrobials and speed up recovery. It must discriminate between viral and parasitic infections too. The efficacy of the process should be established.

Given that hotspots for antimicrobial consumption are not restricted to animal husbandry, efforts in humane acute care hospitals, in particular in dedicated departments like intensive care units [12], require at least as much attention to contain antimicrobial resistance.
